# Covalent and Non-covalent Noble Gas Bonding Interactions in XeF_**n**_ Derivatives (*n* = 2–6): A Combined Theoretical and ICSD Analysis

**DOI:** 10.3389/fchem.2020.00395

**Published:** 2020-05-06

**Authors:** Rosa M. Gomila, Antonio Frontera

**Affiliations:** ^1^Serveis Cientificotècnics, Universitat de les Illes Balears, Palma, Spain; ^2^Department of Chemistry, Universitat de les Illes Balears, Palma, Spain

**Keywords:** σ-hole interactions, π-hole interactions, supramolecular chemistry, inorganic crystal structural database, DFT calculations

## Abstract

A noble gas bond (also known in the literature as aerogen bond) can be defined as the attractive interaction between any element of group-18 acting as a Lewis acid and any electron rich atom of group of atoms, thus following the IUPAC recommendation available for similar π,σ-hole interactions involving elements of groups 17 (halogens) and 16 (chalcogens). A significant difference between noble gas bonding (NgB) and halogen (HaB) or chalcogen (ChB) bonding is that whilst the former is scarcely found in the literature, HaB and ChB are very common and their applications in important fields like catalysis, biochemistry or crystal engineering have exponentially grown in the last decade. This article combines theory and experiment to highlight the importance of non-covalent NgBs in the solid state of several xenon fluorides [XeF_n_]^m+^ were the central oxidation state of Xe varies from +2 to +6 and the number of fluorine atoms varies from n = 2 to 6. The compounds with an odd number of fluorine atoms (*n* = 3 and 5) are cationic (*m* = 1). The Inorganic Crystal Structural Database (ICSD) strongly evidences the relevance of NgBs in the solid state structures of xenon derivatives. The ability of Xe compounds to participate in π,σ-hole interactions has been studied using different types of electron donors (Lewis bases and anions) using DFT calculations (PBE1PBE-D3/def2-TZVP) and the molecular electrostatic potential (MEP) surfaces.

## Introduction

The starting point of the noble gas chemistry was in 1962 with the discovery of XePtF_6_ and XeF_2_ compounds by Bartlett (1962) and Zirin groups (Chernick et al., 1962), respectively. This discovery opened a new field of research that has grown in the last two decades due to the improvements in the experimental techniques and instrumentation to carry out reactions and measurements in extreme conditions (Haner and Schrobilgen, 2015; Grandinetti, 2018). Another interesting step in this field was the synthesis in 2000 by Seidel and Seppelt of the first compound having a noble gas–noble metal bond [AuXe_4_]^2+^ (Seidel and Seppelt, 2000). The formation of an Au–Xe covalent bond itself is counterintuitive if gold is considered as a truly noble metal and xenon a truly noble gas. Nevertheless, after the synthesis and characterization of the XeAuF molecule by Cooke and Gerry (2004), numerous reports have been published in the literature studying the chemistry of Au–Xe–X (X = electron withdrawing group) compounds (Grochala, 2007; Belpassi et al., 2008).

Supramolecular chemistry and molecular recognition (including self-assembly) are intimately related concepts (Busschaert et al., 2015) that rely on the understanding of non-covalent interactions. For instance, chemists working on solid state crystal engineering or solution state supramolecular chemistry aspire to control molecular recognition, designing individual molecules enable to interact with other molecules or themselves conducting the formation of assemblies spontaneously through non-covalent interactions (Schneider, 2009; Desiraju, 2013). The final aim is to control the molecular recognition process precisely to be able to build selective molecular receptors, sensors, supramolecular catalysts, polymers, etc.

A deep understanding of the physical nature of non-covalent interactions (directionality, strength, cooperativity) is essential to dictate supramolecular chemistry processes since they are usually governed by an intricate combination of forces (Schneider and Yatsimirski, 2000). Therefore, a precise description of the non-covalent interactions is essential for the incessant expansion of the supramolecular chemistry. Crystal engineering and molecular recognition commonly trust in moderately strong and directional H-bonding interactions (Desiraju and Steiner, 2001) in combination with less directional but stronger forces like ion pairing. In this sense, charge assisted H-bonds combine strength of an ion-pair and the directionality of dipole dipole interactions. Furthermore, ion–π interactions, either between cations and electron rich π-systems or between anions and acidic rings (Frontera et al., 2011), are also active players in crystal engineering or solution state supramolecular chemistry, including supramolecular catalysis (Zhao et al., 2015). The π-π stacking is another non-covalent interaction that is widely used in molecular recognition and crystal engineering, being particularly relevant in the construction of supramolecular polymers (Meyer et al., 2003).

In addition to the aforementioned conventional interactions, other types of more unconventional interactions where elements of the p-block play the role of hydrogen in H-bonds are gaining importance in supramolecular chemistry (Bauzá et al., 2015; Legon, 2017). Recent advances in host-guest chemistry, catalysis and membrane transport are good examples that illustrate how these interactions are gaining attention. This is particularly true in the fields of crystal engineering and theoretical chemistry, where tetrel (Tr) (Bauzá et al., 2019), pnictogen (Pn) (Scheiner, 2013), chalcogen (Ch) (Scilabra et al., 2019) and halogen bonding (HaB) (Cavallo et al., 2016) are largely utilized and studied. These X–D···A interactions, where X is any atom, D is the σ-hole donor atom (Lewis acid) from groups 13–17 of elements, and A is any electron rich entity (Lewis base) have several common features. The magnitude of the π,σ-hole depends on two factors: (i) the polarizability of D and (ii) the electron withdrawing ability of the X atom. The atomic polarizability increases in a given group on going from lighter to heavier elements. For noble gases (group 18) the polarizability values in atomic units are He = 1.36, Ne = 2.62, Ar = 11.10, Kr = 16.70 and Xe = 27.06 (Bauzá and Frontera, 2020); thus a more intense π,σ-hole is expected for Xe, and, consequently, it is expected to form the strongest interactions.

There are several works and reviews available in the literature where noble gas bonding or aerogen bonding interactions (NgBs) have been studied both experimental and theoretically (Haner and Schrobilgen, 2015; Grandinetti, 2018; Bauzá and Frontera, 2020), which were named as such in 2015 (Bauzá and Frontera, 2015). The purpose of this manuscript is to combine searches on the inorganic crystal structural database (ICSD) and theoretical calculations to explore the ability of XeF_n_ (*n* = 2–6) compounds to form non-covalent NgBs. The theoretical part includes molecular electrostatic potential (MEP) surfaces to identify the directional preference of Xe to participate in NgBs depending on the number of fluorine atoms. Moreover, a set of complexes has been calculated at the PBE1PBE-D3/def2-TZVP level of theory to investigate both the energetic and geometric features of the complexes. The survey of crystal structures retrieved from the ICSD evidences that NgBs between xenon fluorides and lone-pair-possessing atoms are very common.

## Theoretical Methods

The energies of all complexes included in this study were computed at the PBE1PBE-D3/def2-TZVP level of theory. The geometries have been fully optimized imposing either *C*_s_ or *C*_nv_ (*n* = 3,4) symmetry constraints (unless otherwise noted) by using the program Gaussian-16 (Frisch et al., 2016). The interaction energy (or binding energy in this work) ΔE, is defined as the energy difference between the optimized complex and the sum of the energies of the optimized monomers. For the calculations we have used the Weigend def2-TZVP (Weigend and Ahlrichs, 2005; Weigend, 2006) basis set and the PBE1PBE (Adamo and Barone, 1999) DFT functional. The MEP (Molecular Electrostatic Potential) surfaces calculations have been computed at the same level of theory and plotted using the 0.001 a.u. isosurface as the best estimate of the van der Waals surface. The QTAIM formalism has been used to analyse the topology of the electron density (Bader, 1985), using the same level of theory and optimized geometries and making use of the AIMAll program (Keith, 2013). The natural bond orbital (NBO) analysis was performed on some optimized complexes at the same level. The NBO analysis is adequate to study the role of intermolecular orbital interactions or charge transfer in the complexes (Reed et al., 1988). It takes into consideration all possible interactions between filled donor and empty acceptor NBOs and calculating their energetic stabilization by using the second-order perturbation theory. The NBO 3.1 program, as implemented in Gaussian-16 program (Frisch et al., 2016) was used for the calculations.

## Results and Discussion

### XeF_2_

#### X-ray Crystal Structure

**Figure 1:**
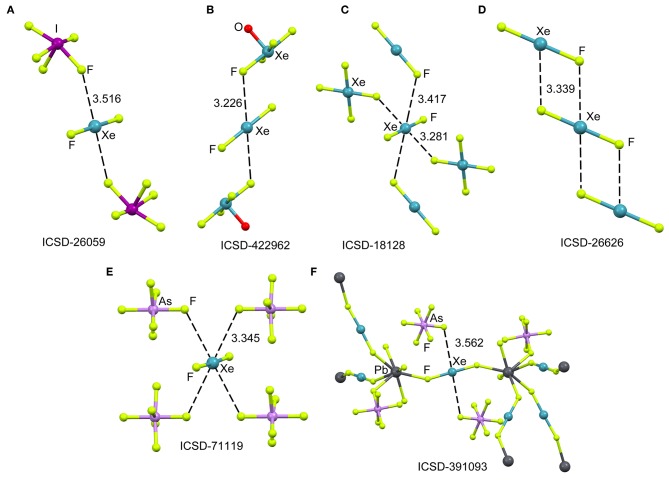
Partial views of the X-ray structures ICSD-26059 **(A)**, ICSD-422962 **(B)**, ICSD-18128 **(C)**, ICSD-26626 **(D)**, ICSD-71119 **(E)**, and ICSD-391093 **(F)**, Distances in Å. NgBs represented as dashed lines.

The ICSD has been inspected manually to investigate the ability of xenon difluoride to participate in NgBs. The sum of van der Waal radii of Xe and F is ΣR_vdw_ = 3.63 Å and the sum of their covalent radii is ΣR_cov_ = 1.97 Å. Figure 1 (top) shows several assemblies retrieved from the X-ray structure of XeF_2_ (Templeton et al., 1963) and three of its cocrystals, i.e., [XeF_2_]·[IF_5_] (Jones et al., 1970), [XeF_2_]·[XeF_4_O] (Hughes et al., 2011) and [XeF_2_]·[XeF_4_] (Burns et al., 1965). It can be observed that all X-ray structures present Xe···F contacts with distances that are clearly longer than ΣR_cov_ and shorter than ΣR_vdw_ thus suggesting the non-covalent nature of these NgBs. Another geometrical aspect that it is worthy to comment is that the F–Xe···F angle is smaller than 90° thus evidencing that the directionality of the NgBs interaction is not strictly perpendicular. This is likely due to the presence of three lone pairs at Xe located perpendicular to the F–Xe–F axis. In the case of [XeF_2_]·[XeF_4_], the Xe establishes four NgBs, two with the XeF_4_ and two with the XeF_2_ (see Figure 1C). The assemblies of [XeF_2_]·[IF_5_], [XeF_2_]·[XeF_4_O], are very similar and both the [IF_5_] and [XeF_4_O] moieties exhibit a square pyramid geometry with one fluorine atom pointing to the Xe (see Figures 1A,B). Finally, the XeF_2_ crystal structure forms self-assembled supramolecular polymers where two symmetrically equivalent Xe···F contacts are established (see Figure 1D).

The XeF_2_ molecule has been also used as a ligand for synthesizing a great variety of coordination compounds. The first compound was isolated in 1991 and it was a silver complex of formula [Ag(XeF_2_)_2_](AsF_6_) (Hagiwara et al., 1991). In the last decade, many coordination complexes have been synthesized using alkaline, alkaline-earth, divalent transition metals, trivalent lanthanides and Pb as the unique element of the p-block (Tavčar and Tramšek, 2015). Several reviews describing coordination compounds with XeF_2_ as a ligand to metal cations of the type [M(XeF_2_)_n_] are available in the literature (Tavčar et al., 2004; Tramšek and Žemva, 2006).

Figure 1 (bottom) shows two examples of XeF_2_ coordination compounds where the Xe participates in NgBs. The X-ray represented in Figure 1E corresponds to a silver compound (Ag ions not shown for clarity) where four symmetrically equivalent Xe···F contacts are formed (Hagiwara et al., 1991). It is expected that the coordination of XeF_2_ to the metal center enhances the ability of Xe to act as Lewis acid. The coordination compound with Pb(II) is shown in Figure 1F, where each XeF_2_ molecule bridges two Pb metal centers, thus generating a 3D coordination polymer (Tramšek et al., 2002). Two Xe···F NgBs are formed with the ${\text{AsF}}_{6}^{-}$ counterions with Xe···F distances that are slightly shorter than ΣR_vdw_.

#### Theoretical Study

**Figure 2:**
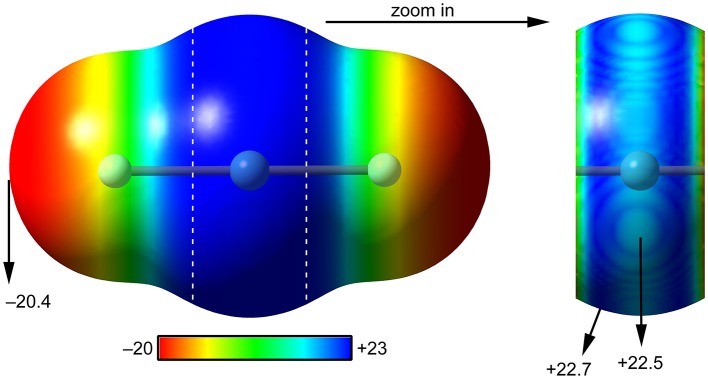
MEP surfaces (0.001 a.u.) of XeF_2_ (left) and a “zoom-in” representation at the PBE1PBE-D3/def2-TZVP level of theory. The MEP energies at selected points are indicated in kcal/mol.

The molecular electrostatic potential (MEP) surface analysis is used herein to rationalize the ability of XeF_2_ to establish NgBs, as illustrated above in Figure 1. The MEP plotted onto the van der Waals surface is useful to know the most electrophilic and nucleophilic parts of the molecule and to rationalize donor-acceptor non-covalent interactions. Figure 2 shows the MEP surface of XeF_2_ and it reveals the existence of a positive belt around the Xe atom and two negative regions at both ends of the molecule (F-atoms). A close examination of the positive belt indicates that the maximum value of MEP is not located strictly perpendicular to the molecular axis at the position of the Ng-atom. Instead they are located in two symmetric belts that are slightly displaced toward the F-atoms (see Figure 2, right). The MEP analysis suggests that Xe(II) molecules should have a strong tendency to establish Ng-bonding interactions with electron rich atoms with some deviation from the perpendicular trajectory.

**Scheme 1:**
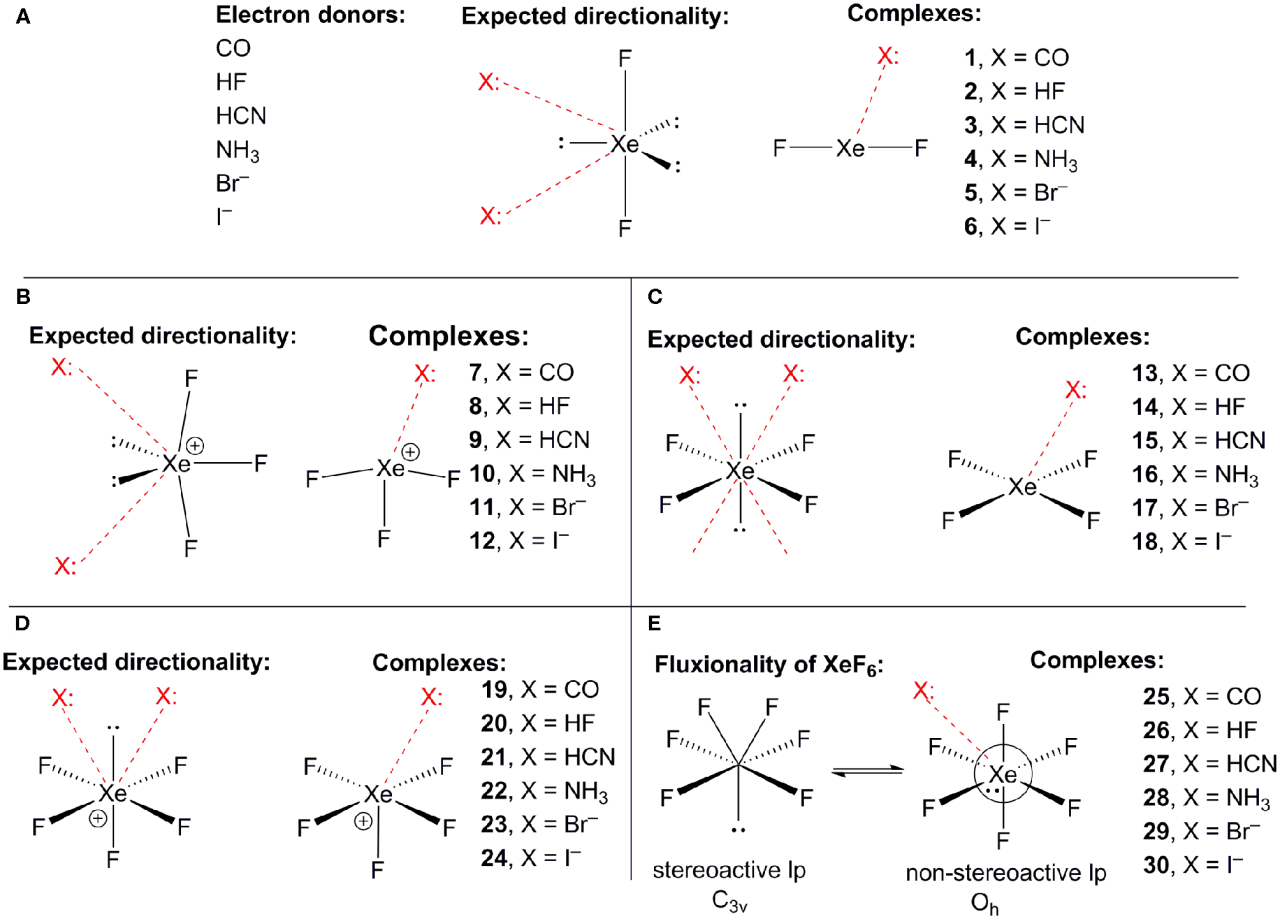
**(A)** Electron donors, expected directionality, location of the stereoactive lone pairs in XeF_2_ and complexes **1**–**6**. **(B)** Expected directionality, location of the stereoactive lone pairs in XeF_3_ and complexes **7**–**12**. **(C)** Expected directionality, location of the stereoactive lone pairs in XeF_4_ and complexes **13**–**18**. **(D)** Expected directionality, location of the stereo-active lone pair in XeF_5_ and complexes **19**–**24**. **(E)** fluxionality of the lone pair in XeF_6_ and complexes **25**–**30**.

Scheme 1A shows the electron donor molecules and complexes of XeF_2_ that have been optimized at the PBE1PBE-D3/def2TZVP. A variety of Lewis bases and anions have been selected to analyze the influence of the basicity and neutral/anion nature of the donor on the interaction energies. We also represent the expected directionality assuming the stereo-active character of the lone pairs and their location is proposed based on the well-known valence-shell electron-pair repulsion (VSEPR) theory, that has been recently revisited (Munárriz et al., 2019).

The interaction energies and distances for complexes **1**–**6** are gathered in Table 1, showing that the interaction energies are favorable in all cases. The energetic results indicate that the CO complex **1** is the weakest one and the Br^−^ the strongest one. In fact, the equilibrium distance of the CO complex is very close to the sum of van der Waals radii whilst the R values for the rest of complexes is much shorter than ΣR_vdw_. As expected, the interaction energies involving the anionic donors are stronger than those with neutral donors, being more favorable for bromide. The interaction energy of complex **4** is moderately strong, in agreement with the stronger basicity of NH_3_ molecule. Finally, it is interesting to highlight that the Xe···F distance computed for the HF complex in in the range of experimental distances observed in the X-ray structures commented above (see Figure 1).

**Table 1:** Interaction energies (ΔE in kcal/mol), F_n_Xe X equilibrium distances (R, Å), sum of van der Waals and covalent radii of interacting atoms (ΣR_vdw_ and ΣR_cov_, Å), electron charge density and total energy density at the bond critical point [ρ(r) and H(r), respectively, in a.u.] at the PBE1PBE-D3/def2-TZVP level of theory for complexes **1** to **30**.

**Complex**	**ΔE**	**R**	**ΣR_**VDW**_**	**ΣR_**COV**_**	**ρ(r)**	**H(r)**
**1** (XeF_2_··CO)	−1.14	3.831	3.86	2.16	0.0044	0.0009
**2** (XeF_2_··FH)	−1.83	3.342	3.63	1.97	0.0064	0.0018
**3** (XeF_2_··NCH)	−2.36	3.547	3.71	2.11	0.0067	0.0012
**4** (XeF_2_··NH_3_)	−4.43	3.361	3.71	2.11	0.0105	0.0009
**5** (XeF_2_··Br) ^−^	−11.59	3.442	4.01	2.60	0.0163	0.0004
**6** (XeF_2_··I) ^−^	−9.10	3.758	4.14	2.79	0.0121	0.0005
**7** (XeF_3_··CO)^+^	−17.44	2.704	3.86	2.16	0.0403	−0.0033
**8** (XeF_3_··FH)^+^	−18.26	2.545	3.63	1.97	0.0333	0.0018
**9** (XeF_3_··NCH)^+^	−35.67	2.438	3.71	2.11	0.0586	−0.0096
**10** (XeF_3_··NH_3_)^+^	−54.66	2.337	3.71	2.11	0.0813	−0.0230
**11** (XeF_3_··Br)	−198.55	2.563	4.01	2.60	0.0831	−0.0248
**12** (XeF_3_··I)	−193.96	2.776	4.14	2.79	0.0682	−0.0230
**13** (XeF_4_··CO)	−2.50	3.539	3.86	2.16	0.0073	0.0010
**14** (XeF_4_··FH)	−4.18	3.159	3.63	1.97	0.0105	0.0020
**15** (XeF_4_··NCH)	−4.29	3.330	3.71	2.11	0.0103	0.0015
**16** (XeF_4_··NH_3_)	−7.02	3.141	3.71	2.11	0.0168	0.0008
**17** (XeF_4_··Br) ^−^	−19.80	3.238	4.01	2.60	0.0240	−0.0004
**18** (XeF_4_··I) ^−^	−15.81	3.530	4.14	2.79	0.0183	0.0001
**19** (XeF_5_··CO)^+^	−10.83	3.010	3.86	2.16	0.0228	0.0004
**20** (XeF_5_··FH)^+^	−16.04	2.675	3.63	1.97	0.0257	0.0032
**21** (XeF_5_··NCH)^+^	−26.73	2.685	3.71	2.11	0.0366	−0.0013
**22** (XeF_5_··NH_3_)^+^	−36.73	2.610	3.71	2.11	0.0508	−0.0070
**23** (XeF_5_··Br)	−179.14	2.585	4.01	2.60	0.0816	−0.0233
**24** (XeF_5_··I)	−174.20	2.798	4.14	2.79	0.0673	−0.0172
**25** (XeF_6_··CO)	−3.47	3.162	3.86	2.16	0.0149	0.0012
**26** (XeF_6_··FH)	−3.47	2.964	3.63	1.97	0.0116	0.0029
**27** (XeF_6_··NCH)	−6.92	2.870	3.71	2.11	0.0231	0.0012
**28** (XeF_6_··NH_3_)	−18.36	2.586	3.71	2.11	0.0502	−0.0069
**29** (XeF_6_··Br) ^−^	−43.29	2.807	4.01	2.60	0.0556	−0.0098
**30** (XeF_6_··I) ^−^	−36.75	3.050	4.14	2.79	0.0449	−0.0066

**Figure 3:**
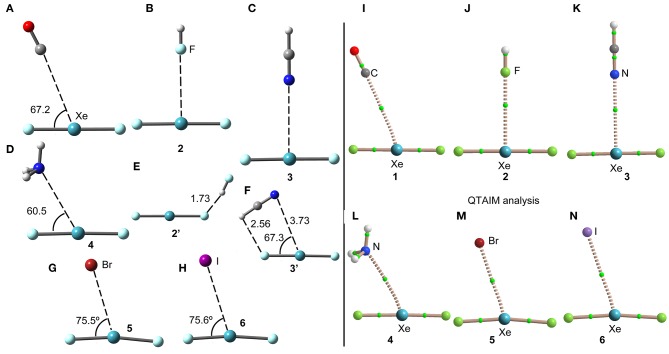
Left panel: PBE1PBE-D3/def2-TZVP Optimized geometries of complexes **1 (A)**, **2 (B)**, **3 (C)**, **4 (D)**, **2' (E)**, **3' (F)**, **5 (G)** and **6 (H)**. Distances in Å. Right panel: QTAIM distribution of bond critical points (green spheres) and bond paths for complexes **1 (I)**, **2 (J)**, **3 (K)**, **4 (L)**, **5 (M)**, and **6 (N)** at the PBE1PBE-D3/def2-TZVP level of theory.

The geometries of the XeF_2_ complexes are given in Figure 3 (left panel), where it can be observed that the directionality of their NgB interaction agrees well with the expectation derived from the VSEPR theory and also the MEP surface represented in Figure 3. The X:···Xe–F angle varies from 60 to 75°. In the stronger anionic complexes **5** and **6**, the XeF_2_ molecule bents as a consequence of the formation of strong NgBs. In complexes **2** and **3**, where an acidic proton is present in the electron donor molecule, the optimization of the complexes using *C*_s_ symmetry yields either a H-bonded complex in case of HF (see Figure 3E) or a combination of HB and NgB interactions in case of HCN (see Figure 3F). In order to estimate the energies associated to the NgBs in these complexes without the contribution of the HBs, optimizations imposing *C*_2v_ symmetry (Figures 3B,C) have been performed and only the interaction energies corresponding to the *C*_2v_ geometries are given in Table 1.

The NgB interaction in complexes **1**-**6** has been characterized using the quantum theory of “atoms-in-molecules” (QTAIM) (Bader, 1985). For all complexes the NgB is characterized by a bond critical point (CP) and bond path connecting the electron rich atom to the Xe (see Figure 3, right panel). The values of electron charge density ρ(r) at the bond CPs are tabulated in Table 1. Interestingly, the values of ρ(r) at the bond CPs that characterize the NgB correlate well with the interaction energies by using a logarithmic fitting (regression coefficient, *r* = 0.972, see Supplementary Material) as previously described in the literature (Bader, 1990). Therefore, the value of ρ(r) at the bond CP can be used as a measure of the strength of the NgB interaction. The values of the total energy density [H(r)] at the bond CPs are also summarized in Table 1 since they are adequate to differentiate covalent and non-covalent interactions. Positive values of H(r) indicate non-covalent bonding, negative and small values of H(r) are indicative of partial covalent character, and large and negative values of H(r) along with large values of ρ(r) designate covalent bonding (Bader et al., 1987; Bader, 1990). The examination of the values of H(r) in Table 1 evidences the non-covalent nature of the interaction in all complexes.

As exemplifying system, we have selected the complex with NH_3_ to perform the NBO analysis. This type of study is adequate to analyse the importance of orbital donor-acceptor interactions. In the XeF_2_···NH_3_ system, we have found a modest donor-acceptor interaction from the lone pair orbital of N to the Xe–F antibonding orbital [LP(n) → σ^*^(Xe–F)] with a concomitant stabilization energy of *E*^(2)^ = 1.02 kcal/mol. Although the orbital contribution is small, it is not negligible compared to the total interaction energy (~25%).

### ${\text{XeF}}_{3}^{+}$

#### X-ray Crystal Structures

At the beginning of the development of noble gas chemistry, several adducts of XeF_2_ and XeF_6_ with strong fluoride ion acceptor molecules were synthesized (Holloway, 1968; Sladky et al., 1969). Moreover, several works (Edwards et al., 1963; Cohen and Peacock, 1966; Bartlett and Sladky, 1968) tried to synthesize XeF_4_ adducts in combination to fluoride acceptors like SbF_5_, TaF_5_, AsF_5_ etc. without success. In fact, instead to forming the adducts, the [XeF_3_]^+^ cation is generated, for instance by simply dissolving XeF_4_ or XeF_2_/XeF_4_ in SbF_5_.

**Figure 4:**
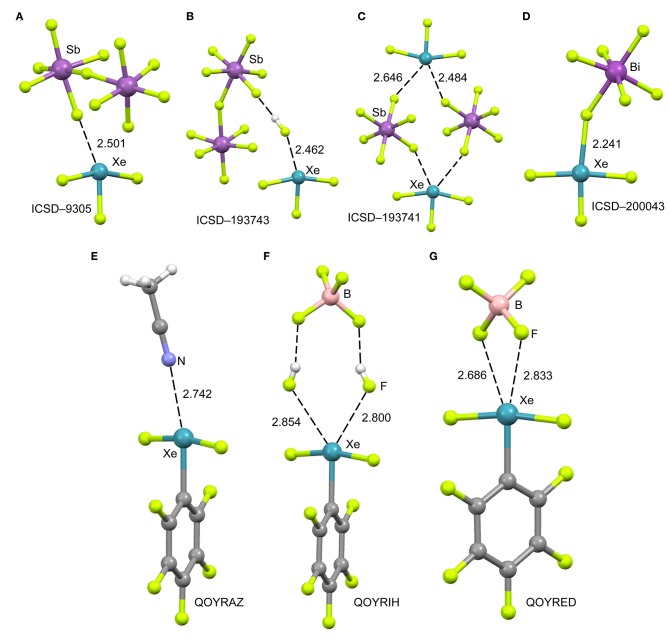
Top panel: Partial views of the X-ray structures ICSD-9305 **(A)**, ICSD-193743 **(B)**, ICSD-193741 **(C)** and ICSD-200043 **(D)**. Distances in Å. NgBs represented as dashed lines. Bottom panel: Partial views of the X-ray structures with Cambridge Structural Database reference codes QOYRAZ **(E)**, QOYRIH **(F)** and QOYRED **(G)**. Distances in Å. NgBs represented as dashed lines.

The cationic nature of xenon trifluoride, anticipates a strong binding with electron rich atoms due to the strong contribution of electrostatic forces (charge-charge or charge-dipole). In Figure 4 (top panel), several X-ray structures are represented to illustrate the characteristics of the Xe···F bonds in [XeF_3_]^+^ salts. The X-ray structure of the [XeF_3_]^+^[Sb_2_F_11_]^−^ salt (Figure 4A) (McKee et al., 1973) shows a short contact between one F-atom of the anion and the Xe-atom that exhibits the typical T-shaped geometry. It is worth mentioning that the F-atom of the anion that makes the short contact is in the same plane defined by the four atoms of the XeF_3_ cation. Although the contact is significantly shorter than ΣR_vdw_, (indicating some degree of covalency), the two lone pairs located at the Xe(IV) atom are not involved in the bonding since they are not located in the molecular plane. It is interesting to comment the structure ICSD-193743 that has the following formula [H_5_F_4_][SbF_6_]·2[XeF_3_·HF][Sb_2_F_11_], thus including HF units in the structure (Brock et al., 2013). In Figure 4B only the [XeF_3_·HF][Sb_2_F_11_] fragment is represented, where the H-atom has been added in an arbitrary position. Again the interacting F-atom of the HF is located in the molecular plane and establishes a very short NgBs with the Xe-atom. It is also remarkable the solid state structure of the [XeF_3_]^+^[SbF_6_]^−^ salt that forms tetrameric assemblies in the solid state where two different Xe···F NgB contacts are established (Brock et al., 2013). A common feature of all X-ray structures presented in Figures 4A–C is that the electron rich atom is not located exactly opposite to the F–Xe bond, in fact the F_ax_-Xe···F angle varies from 154 to 160° in these salts. Interestingly, if [BiF_6_]^−^ is used as anion instead of [SbF_6_]^−^ (see Figure 4D) (Gillespie et al., 1977), the Xe····F bond becomes very short (close to ΣR_cov_) and the F–Xe···F is close to linearity, thus suggesting the formation of a partial covalent bond. The approximation of the fluoride lone-pair to the middle of the edge of the trigonal bipyramid containing the two stereo-active lone pairs, forces the geometry around the Xe to be approximately square-planar. Thus the overall stereochemistry changes from a T-shaped AX_3_E_2_ in the [XeF_3_]^+^[BiF_6_]^−^ salt to a square-planar AX_4_E_2_ structure in the [XeF_3_]^+^[SbF_6_]^−^ salt. This behavior agrees well with the low acidity of BiF_5_ molecule compared to SbF_5_ (Gillespie and Pez, 1969).

In 2014 the synthesis and X-ray characterization of several [C_6_F_5_XeF_2_]^+^ salts were published (Koppe et al., 2014). The ligand arrangement around xenon in the three salts shown in Figure 4 (bottom panel) is T-shaped, in accordance with the expected arrangement of three bonding electron pairs and two additional electron lone pairs in the xenon valence shell. The electron lone pairs cause the F–Xe(I)–F angles to bend toward the C_6_F_5_ group producing nonlinear F–Xe(I)–F angles (~170°). The distances of the NgB contacts are longer in these salts compared to the [XeF_3_]^+^ salts because the C_6_F_5_ group (Xe–C bond) is less electron withdrawing than fluorine atom (Xe–F bond). Again the electron donor atom is not located exactly opposite to the Xe–C bond, as further commented below (DFT study). It is interesting to highlight the QOYRIH structure (see Figure 4F) where two HF molecules connect the anion and cation by establishing two Xe···F NgBs with the Xe atom and two F–H···F H-bonds with the [BF_4_]^−^ anion. In the [C_6_F_5_XeF_2_]^+^ [BF_4_]^−^ salt (Figure 4G), the anion establishes two NgBs with the counter-cation. In spite the NgB contacts in [C_6_F_5_XeF_2_]^+^ salts are longer than those in [XeF_3_]^+^ salts, the distances are significantly shorter than ΣR_vdw_, due to the electrostatic attraction between the counterions.

#### DFT Calculations

**Figure 5:**
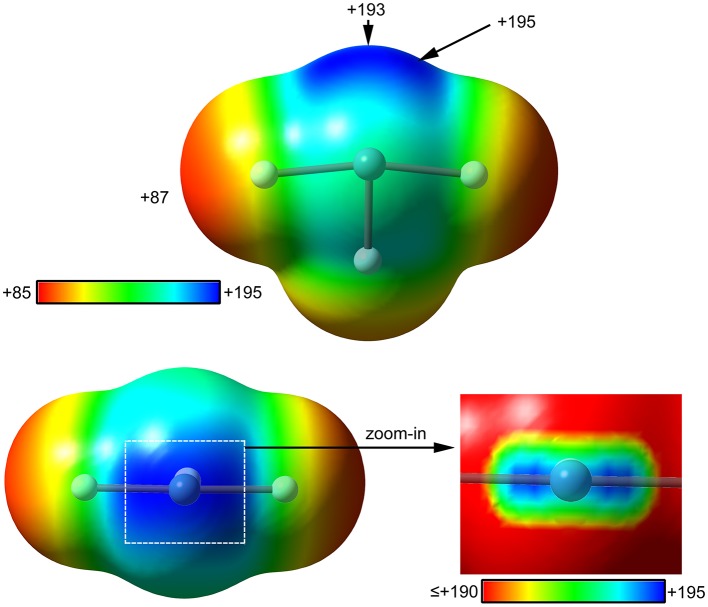
MEP surfaces (0.001 a.u.) of ${\text{XeF}}_{3}^{+}$ (top and bottom-left) and a “zoom-in” representation at the PBE1PBE-D3/def2-TZVP level of theory. The MEP energies at selected points are indicated in kcal/mol.

The molecular electrostatic potential (MEP) surface analysis of [XeF_3_]^+^ cation has been computed to rationalize its ability to establish charge assisted NgBs, as shown in the X-ray structures represented Figure 4. Figure 5 shows the MEP surfaces of [XeF_3_]^+^ using two different orientations and it reveals the existence of a positive region at the Xe atom and opposite to the equatorial F-atom (see Scheme 1B). A close examination of the positive σ-hole shows that the maximum value of MEP is not located strictly along the extension of the Xe–F bond. Instead there are two symmetric σ-holes that are slightly displaced toward the axial F-atoms (see Figure 5, bottom-right). The MEP analysis agrees well with the directionality of the NgBs observed in the aforementioned X-ray structures.

The same electron donors used above for XeF_2_ complexes (see Scheme 1A) have been also used for the theoretical study of the [XeF_3_]^+^ cation. The structure of [XeF_3_]^+^ is T-shaped with *C*_2v_ symmetry and it is derived from a trigonal bipyramid with two stereo-active lone pairs occupying the equatorial positions with a Xe in the +4 oxidation state (see Scheme 1B). Taking into consideration the location of the lone pairs the most favorable approximation of an electron rich atom should avoid the spatial region of these lone pairs. Thus, the expected directionality of the NgB interaction is indicated by the red dashed lines in Scheme 1 and agrees well with and the position of the σ-holes revealed by the MEP surface.

The interaction energies and distances for complexes **7**–**12** are gathered in Table 1. It can be observed that the interaction energies are very large in all cases, as expected taking into consideration the cationic nature of the electron acceptor. Complexes **7** and **8** are the weakest ones and present equilibrium distances that are ~0.5 Å longer than the sum of their covalent radii (also tabulated in Table 1). The equilibrium distances of complexes **9** and **10** are slightly longer (0.2–0.3 Å) than ΣR_cov_ thus indicative of partial covalency, especially in the NH_3_ complex **10**. Finally, the equilibrium distance of anionic complexes **11** and **12** is very similar to their ΣR_cov_ thus suggesting the formation of a covalent bond. In fact, the binding energies computed for these complexes are very large (< -193 kcal/mol) due to the covalent nature of the bond.

**Figure 6:**
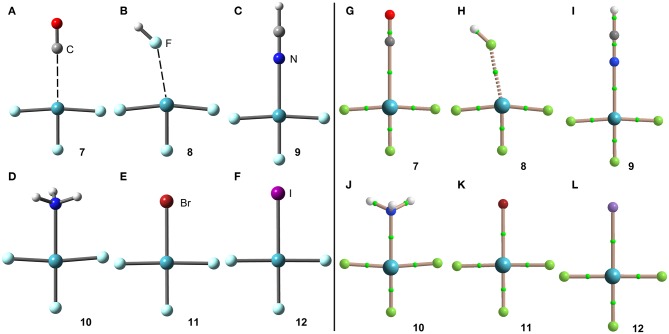
Left panel: PBE1PBE-D3/def2-TZVP Optimized geometries of complexes **7 (A)**, **8 (B)**, **9 (C)**, **10 (D)**, **11 (E)** and **12 (F)**. Distances in Table 1. Right panel: QTAIM distribution of bond critical points (green spheres) and bond paths for complexes **7 (G)**, **8 (H)**, **9 (I)**, **10 (J)**, **11 (K)**, and **12 (L)** at the PBE1PBE-D3/def2-TZVP level of theory.

The geometries of the [XeF_3_]^+^ complexes are given in Figure 6 (left panel), where it can be observed that for most of the complexes the electron rich atom is located along the extension of the Xe–F bond, yielding to the typical square planar geometry of XeX_4_E_2_ compounds with the stereo-active lone pairs pointing to the axial positions (Haner and Schrobilgen, 2015). This fact confirms the great degree of covalency in [XeF_3_]^+^ complexes. Only the complex with HF follows the expected orientation, also in good agreement with the X-ray structures involving HF as electron donor (see Figures 4B,F). It is surprising the location of the CO in complex **7**, exactly opposite to the Xe–F_eq_ bond, due to the apparent non-covalent nature of the NgB interaction in this complex.

The NgB covalent/non-covalent nature of the interaction in complexes **7**-**12** has been unveiled by using the quantum theory of “atoms-in-molecules” (QTAIM) (Bader, 1985). Similarly to XeF_2_ complexes, the NgB in [XeF_3_]^+^ complexes are characterized by a bond critical point (CP) and bond path interconnecting the electron rich and Xe atoms (see Figure 6, right panel). The values of electron charge density ρ(r) at the bond CPs are listed in Table 1. They are significantly larger than those observed in complexes **1**–**6**, in line with the stronger interaction. For this set of complexes, the logarithmic fitting [ρ(r) vs ΔE] shows a modest relationship with a regression coefficient of *r* = 0.818, see Supplementary Material. The values of the total energy density [H(r)] at the bond CPs summarized in Table 1 are indicative of partial covalent character in all complexes apart from complex **8**, in good agreement with the geometric features of the complexes. Surprisingly, the CO complex also exhibits a covalent character [H(r) = −0.0033 a.u.], which is probably due to the fact that the equilibrium distance (2.704 Å) is more than 1 Å shorted than ΣR_vdw_ (3.86 Å).

The covalent character of these complexes is also confirmed by the NBO analysis. Again, using the (XeF_3_··NH_3_)^+^ as model complex, the NBO treats the N–Xe bond as covalent since the energetic contribution of the orbital [LP(n) → σ^*^(Xe–F)] donor-acceptor interaction is −73.14 kcal/mol, significantly stronger that the interaction energy (see Table 1).

### XeF_4_

#### X-ray Crystal Structures

**Figure 7:**
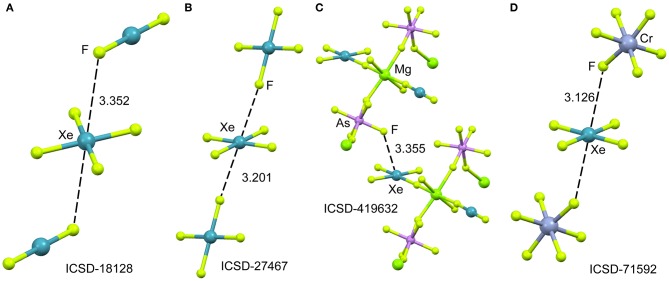
Partial views of the X-ray structures ICSD-18128 **(A)**, ICSD-27467 **(B)**, ICSD-419632 **(C)** and ICSD-71592 **(D)**. Distances in Å. NgBs represented as dashed lines.

In spite of XeF_4_ was the first fluoride of xenon to be discovered, it is the most difficult to synthesize among the series of binary xenon fluorides (XeF_2_, XeF_4_, and XeF_6_). There is a few number of X-ray structures including the XeF_4_ moiety and they are represented in Figure 7. One of them is the XeF_2_·XeF_4_ adduct (Burns et al., 1965) already described above from the perspective of XeF_2_ as NgB donor. In this section, the X-ray structure is analyzed from the opposite point of view, that is considering XeF_4_ as electron acceptor and XeF_2_ as electron donor. The XeF_4_ participates in two short Xe···F contacts with the adjacent XeF_2_ molecules, establishing two symmetrically equivalent NgBs (see Figure 7A). A similar arrangement is observed in the X-ray structure of XeF_4_ (Ibers and Hamilton, 1963), where the central Xe atom participates in two NgBs above and below the molecular plane (see Figure 7B). In contrast to the behavior of XeF_2_, coordination compounds involving XeF_4_ acts ligand are scarce in the literature due to lower fluorobasicity of XeF_4_. One example is given in Figure 7C (Tavčar and Žemva, 2009), where it is coordinated to Mg(II) and, simultaneously, establishes a NgB interaction with the adjacent (also coordinated) ${\text{AsF}}_{6}^{-}$ anion. A partial view of the X-ray structure ([XeF_5_][CrF_5_])_4_·XeF_4_ adduct is shown in Figure 7D (Lutar et al., 1992), where the XeF_5_ units have been omitted for clarity. The distorted CrF_6_-octahedra are connected to each other via Xe···F bridging NgBs. The stereo-active electron lone pairs lie above and below the XeF_4_-plane, preventing the approximation of the electron rich atom along the *C*_4_ axis. Therefore, in all X-ray structures gathered in Figure 7, the approach occurs between the lone pairs and the molecular plane to minimize the repulsions between the lone pairs of Xe and the electron rich atom.

#### DFT Calculations

**Figure 8:**
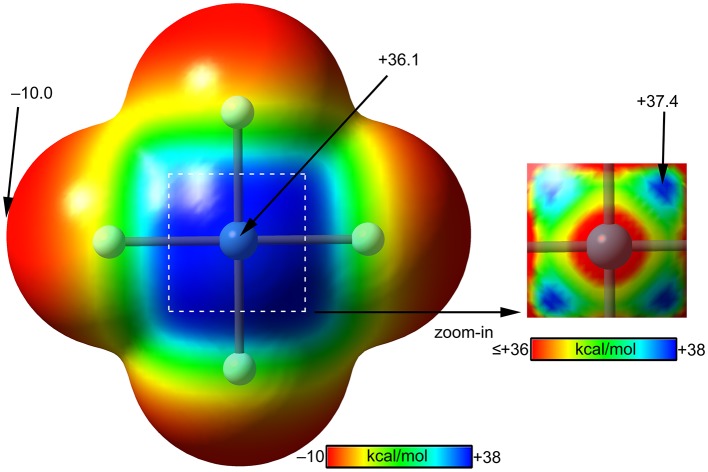
MEP surfaces (0.001 a.u.) of XeF_4_ (left) and a “zoom-in” representation at the PBE1PBE-D3/def2-TZVP level of theory. The MEP energies at selected points are indicated in kcal/mol.

The molecular electrostatic potential (MEP) surface of [XeF_4_] is represented in Figure 8. The minimum MEP is located at the F-atoms and, remarkably, the value (−10 kcal/mol) is half the one of XeF_2_, confirming the less fluorobasicity of this molecule and explaining the weak ability of this molecule as coordination ligand. The MEP surface plot also shows a large π-hole located at the Xe-atom above and below the molecular plane. A close examination of the positive region reveals the existence of four symmetric π-holes that are displaced toward the bisectrix of the F–Xe–F angle (see Figure 8, right). The MEP analysis strongly agrees with the directionality of the NgBs observed in the X-ray structures represented in Figure 5. The MEP maximum in XeF_4_ is significantly larger than that in XeF_2_, thus stronger NgB interactions are expected.

Using the same set of electron donors the energetic and geometric features of XeF_4_ complexes have been studied, as indicated in Scheme 1C. The structure of XeF_4_ is square planar with D_4h_ symmetry and it is derived from an octahedral geometry with two stereo-active lone pairs occupying the axial positions with a Xe in the +4 oxidation state (see Scheme 1C). Taking into consideration the location of the lone pairs the most favorable approximation of an electron rich atom should avoid the spatial region of these lone pairs, as aforementioned. Thus, the expected directionality of the NgB interaction is indicated by the red dashed lines in Scheme 1C and agrees well with and the position of the four π-holes revealed by the MEP surface.

The interaction energies and equilibrium distances of NgB complexes **13**–**18** are summarized in Table 1. It can be observed that the NgB interaction energies are stronger in XeF_4_ complexes than those in XeF_2_ complexes, as predicted by the MEP analysis. Similarly, to the behavior of XeF_2_, complexes **13** (X = CO), **14** (X = HF) and **15** (X = HCN) are the weakest ones. All complexes exhibit equilibrium distances that are shorter than ΣR_vdw_ and significantly longer than ΣR_cov_ thus suggesting the non-covalent nature of the interaction. As expected, the most favorable neutral complex corresponds to the ammonia (**16**) and the anionic complexes **17** and **18** present the stronger interactions of this series.

**Figure 9:**
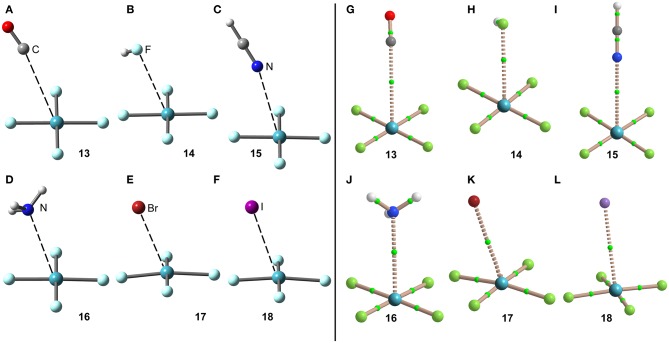
Left panel: PBE1PBE-D3/def2-TZVP Optimized geometries of complexes **13 (A)**, **14 (B)**, **15 (C)**, **16 (D)**, **17 (E)** and **18 (F)**. Distances in Å. Right panel: QTAIM distribution of bond critical points (green spheres) and bond paths for complexes **13 (G)**, **14 (H)**, **15 (I)**, **16 (J)**, **17 (K)**, and **18 (L)** at the PBE1PBE-D3/def2-TZVP level of theory.

The optimized geometries of the XeF_4_ complexes are given in Figure 9, left panel, where it can be observed that the electron rich atom in complexes **13**–**16** is located over the bisector of the F–Xe–F bond at distances that range from 3.1 to 3.6 Å (see Table 1), in good agreement with the X-ray structures and MEP surface. It should be mentioned that the optimization of anionic complexes has been performed imposing *C*_s_ symmetry and locating the anion over one Xe–F bond. In case it is located over the bisector, the optimization yields to the nucleophilic attack of the anion to the Xe-atom, yielding a planar and pentacoordinated [XeF_4_X]^−^ anion (X = Br, I). This result agrees well with the X-ray structure of the [XeF_5_]^−^ anion that is planar (~D_5h_-geometry) (Christe et al., 1991).

The NgB interaction in complexes **13**–**18** has been further characterized using the QTAIM analysis. In agreement with previous observations, the NgB is characterized by a bond critical point (CP) and bond path that connects the electron rich atom to the Xe (see Figure 9, right panel). The values of electron charge density ρ(r) at the bond CPs are tabulated in Table 1 and analogously to XeF_2_ complexes the values of ρ(r) at the bond CPs that characterize the NgB correlate remarkably well with the interaction energies by using a logarithmic fitting (regression coefficient, *r* = 0.965, see Supplementary Material), thus confirming that the value of ρ(r) at the bond CP can be used as a measure of the strength of the NgB interaction. The values of the total energy density [H(r)] at the bond CPs are also summarized in Table 1, which corroborate the non-covalent nature of the interaction in all complexes. Only the Br^−^ complex exhibit some covalent character as deduced by its negative and small H(r) value and strong binding energy.

The NBO analysis has been carried out for the XeF_4_···NH_3_ complex and the orbital interaction is similar to the XeF_2_···NH_3_ complex with a LP(N) → σ^*^(Xe–F) interaction of *E*^(2)^ = 1.12 kcal/mol, however it is smaller compared to the total interaction energy (~14%). Therefore the interaction is clearly dominated by electrostatic effects.

### ${\text{XeF}}_{5}^{+}$

#### X-ray Crystal Structures

**Figure 10:**
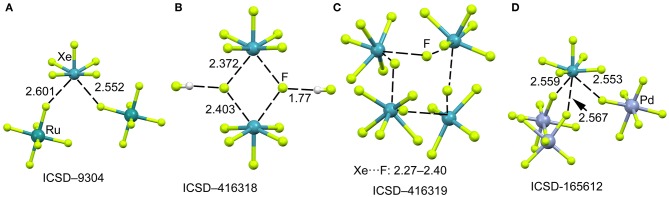
Partial views of the X-ray structures ICSD-9304 **(A)**, ICSD-416318 **(B)**, ICSD-416319 **(C)** and ICSD-165612 **(D)**. Distances in Å. NgBs represented as dashed lines.

The mixture of XeF_6_ and RuF_5_ yields the [XeF_5_]^+^[RuF_6_]^−^ salt, as represented in Figure 10A (Christe et al., 1991). The structural analysis shows that each xenon atom is bonded to five fluorine atoms in an approximately square-pyramidal arrangement. Each ruthenium atom is surrounded by six fluorine atoms in an octahedral coordination mode. The xenon atom in [XeF_5_]^+^ cation retains an stereo-active lone pair, therefore it can be assumed that it is pseudooctahedrally coordinated with five F atoms and the sterically active valence-electron pair that is located along the fourfold axis. Therefore, the approximation of any electron rich atom is expected to be below the basal plane of the [XeF_5_]^+^ cation and off axis, as observed in the crystal structures represented in Figure 10. In the particular case of [XeF_5_]^+^[RuF_6_]^−^ salt, the Xe atom establishes two NgB contacts with two F-atoms of the counterions that are located below the F–Xe–F bisector.

When XeF_6_ is crystallized from anhydrous HF, an interesting compound is obtained that corresponds to the formulae ([XeF_5_]^+^)_2_·([HF_2_]^−^)_2_·HF (Hoyer et al., 2006). The most interesting feature observed in the solid state of this structure is the existence of dimeric units of [XeF_5_]^+^[F]^−^ (see Figure 10B) that are stabilized by the formation of four Xe···F contacts. The [XeF_5_]^+^[F]^−^ dimer also interacts with two HF molecules by H-bonding interactions. The same type of dimers has been also obtained without the co-crystalized solvent molecules upon recrystallization using CF_2_Cl_2_. It is also interesting to highlight the product (see Figure 10C) that is obtained by recrystallization from inert solvents at low temperature. It is a regular tetrameric unit ([XeF_5_]^+^·F^−^)_4_ formed by four square pyramidal [XeF_5_]^+^· that are connected by four Xe···F···Xe bridges with similar distances and angles (118–121°). Figure 10D shows a partial view of the X-ray structure of [XeF_5_${]}_{2}^{+}\cdot$[PdF_6_]^2−^ salt (Lutar et al., 1998). It can be observed that each [XeF_5_]^+^·cation establishes three charge assisted NgBs with the surrounding [PdF_6_]^2−^ units. In general the Xe···F distances in the four X-ray structures shown in Figure 10 are shorter than those preciously described for the [XeF_3_]^+^ cation, thus suggesting stronger binding and higher covalency.

#### DFT Calculations

**Figure 11:**
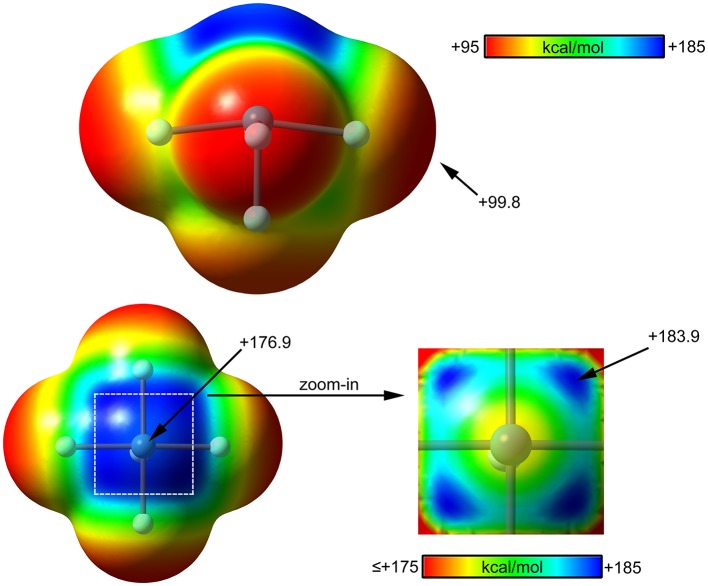
MEP surfaces (0.001 a.u.) of ${\text{XeF}}_{5}^{+}$ (top and bottom-left) and a “zoom-in” representation at the PBE1PBE-D3/def2-TZVP level of theory. The MEP energies at selected points are indicated in kcal/mol.

The molecular electrostatic potential (MEP) surface analysis of [XeF_5_]^+^ cation has been computed to rationalize its ability to establish charge assisted NgBs. Figure 11 shows the MEP surfaces of [XeF_5_]^+^ using two different orientations and it reveals the existence of a large and positive region at the Xe atom and opposite to the axial F-atom. A close examination of the positive σ-hole shows that the maximum value of MEP is not located strictly along the extension of the Xe–F bond. Instead there are four symmetric σ-holes that are slightly displaced toward the bisectors of the F–Xe–F (F atoms in *cis*, see Figure 11, bottom-right), similarly to the behavior described above for the neutral XeF_4_. The MEP analysis strongly agrees with directionality of the NgBs observed in the X-ray structures shown in Figure 10.

The computed [XeF_5_]^+^ complexes are shown in Scheme 1D where the geometry of [XeF_5_]^+^ is square-pyramidal with *C*_4v_ symmetry that derives from a pseudooctahedral with the stereo-active lone pair occupying the remaining axial position with a Xe in the +6 oxidation state (see Scheme 1D). Taking into consideration the location of this lone pair the most favorable approximation of an electron rich atom should avoid the spatial region occupied by this lone pair, as depicted using red dashed lines.

**Figure 12:**
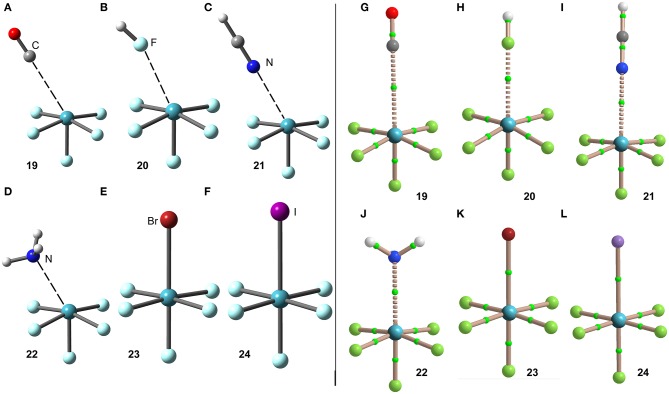
Left panel: PBE1PBE-D3/def2-TZVP Optimized geometries of complexes **19 (A)**, **20 (B)**, **21 (C)**, **22 (D)**, **23 (E)** and **24 (F)**. See Table 1 for distances. Right panel: QTAIM distribution of bond critical points (green spheres) and bond paths for complexes **19 (G)**, **20 (H)**, **21 (I)**, **22 (J)**, **23 (K)**, and **24 (L)** at the PBE1PBE-D3/def2-TZVP level of theory.

The interaction energies and distances for complexes **19**–**24** are gathered in Table 1. It can be observed that the interaction energies are larger than those of XeF_4_ in all cases, as expected taking into consideration the cationic nature of the electron acceptor. Complexes **19** and **20** are the weakest ones and present equilibrium distances that are longer than the sum of their covalent radii (also tabulated in Table 1). Complexes **21** and **22** exhibit moderately strong binding energies and equilibrium distances that are 0.5 Å longer than ΣR_cov_. Taken together, these results suggest a partial covalency of the NgB in these complexes. Interestingly, Figure 12 (left panel) shows that in all complexes with neutral electron donors the electron rich atom points to one of the four σ-holes described in Figure 11. This behavior is opposite to the previously described for the [XeF_3_]^+^ complexes, where all electron rich atoms were located opposite to the Xe–F_eq_ bond apart from the HF complex. Finally, the equilibrium distance of anionic complexes **23** and **24** is very similar to their ΣR_cov_ thus suggesting the formation of a pure covalent bond. In fact, the interaction energies for these complexes are very large and the geometry around the Xe atom is octahedral.

The NgB covalent/ non-covalent nature of the interaction in complexes **19**–**24** has been analyzed by using the quantum theory of “atoms-in-molecules” (QTAIM). Similarly to the rest of NgB complexes of XeF_2_, [XeF_3_]^+^ and XeF_4_, the NgB in [XeF_5_]^+^ complexes is characterized by a bond critical point (CP) and bond path interconnecting the electron rich and Xe atoms (see Figure 12, right panel). The values of electron charge density ρ(r) at the bond CPs are listed in Table 1 and they are significantly larger than those observed in complexes **13**–**18**, and similar to those of complexes **7**–**12**. For this set of complexes, the value of ρ(r) at the bond CP also correlates well with the interaction energy, since the logarithmic fitting gives a regression coefficient of *r* = 0.973, see Supplementary Material. The values of the total energy density [H(r)] at the bond CPs summarized in Table 1 are indicative of partial covalent character in complexes **21** and **22**, in good agreement with the energetic features of these complexes. The H(r) values also confirm the covalent nature of the NgBs in complexes **23** and **24**, in line with the covalent distances and strong binding energies.

The NBO analysis of complex **22** (X = NH_3_) shows a moderately strong orbital donor acceptor interaction [LP(N)–σ^*^(Xe–F)] with an associated stabilization energy of *E*^(2)^ = −11.5 kcal/mol. This result agrees well with the QTAIM analysis that anticipated partial covalent character [small and negative H(r)]. In fact, the orbital contribution accounts for the 31% of the total interaction energy.

### XeF_6_

#### X-ray Crystal Structures

It has been recently reported (Matsumoto et al., 2015) the syntheses and X-ray characterization of two adducts of XeF_6_ with acetonitrile of composition F_6_Xe(NCCH_3_) and F_6_Xe(NCCH_3_)_2_·CH_3_CN. They are good examples of σ-hole NgB interactions and are the first X-ray structures where the electron donor is a nitrogen atom. In the F_6_Xe(NCCH_3_), the XeF_6_ unit presented a *C*_3v_ symmetry similar to that proposed for the gas-phase XeF_6_. Other studies have shown that the NgBs in these systems are predominantly electrostatic in nature (Haner et al., 2016).

**Figure 13:**
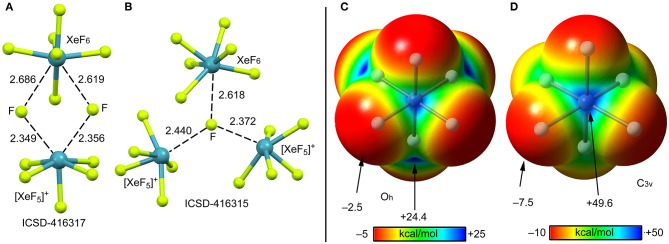
Left panel: Partial views of the X-ray structures ICSD-416317 **(A)** and ICSD-416315 **(B)**. Distances in Å. NgBs represented as dashed lines. Right panel: MEP surfaces (0.001 a.u.) of octahedral **(C)** and C_3v_ **(D)** XeF_6_ at the PBE1PBE-D3/def2-TZVP level of theory. The MEP energies at selected points are indicated in kcal/mol.

According to several experimental techniques including crystal X-ray diffraction and neutron powder diffraction, among others, XeF_6_ exists in at least six different modifications, depending on the temperature (Hoyer et al., 2006). At high temperature XeF_6_ forms a tetramer, better described as (${\text{XeF}}_{5}^{+}$F^−^)_3_·XeF_6_ assembly. A partial view of this tetramer is represented in Figure 13A where only two fluoride anions, one [XeF_5_]^+^ cation and the XeF_6_ unit have been represented for clarity. It can be observed that the fluoride anions bridge the [XeF_5_]^+^ cation and the XeF_6_ units by means of four NgBs. Those involving the cation are shorter than those involving the neutral XeF_6_ that maintains a pseudoctahedral geometry. Figure 13B shows the other form of XeF_6_ that is stable at high temperature (obtained by sublimation of the other one). The structure is also tetrameric and better described as (${\text{XeF}}_{5}^{+}$F^−^)_3_·XeF_6_ assembly. In this case the fluoride anion is stabilized by three NgBs, one with the XeF_6_ unit and two with the [XeF_5_]^+^ cation. Again, the NgB distances involving the XeF_6_ unit are longer than those with [XeF_5_]^+^ cation. In this X-ray structure the geometry of the XeF_6_ unit is approximately *C*_3v_.

#### DFT Calculations

The molecular electrostatic potential (MEP) surface of [XeF_6_] is represented in Figures 13 (right panel) using the octahedral (**C**) and *C*_3v_ (**D**) symmetries. The MEP value at the F-atoms is −2.5 kcal/mol in the octahedral form and −7.5 kcal/mol in the *C*_3v_ form, thus revealing a very low fluorobasicity. For the octahedral XeF_6_, the MEP surface plot also shows six symmetrically equivalent σ-holes (24.4 kcal/mol) located in the middle of the six octahedral faces. In contrast, for the *C*_3v_ form of XeF_6_, the maximum value of MEP is more than twice the value obtained for the octahedral form (+49.6 kcal/mol) and it is located at one face of the polyhedron.

The minimum structure of XeF_6_ is still under discussion (Kaupp et al., 1996; Seppelt, 2015; Gawrilow et al., 2018; Zhao et al., 2019), since most of the theoretical methods suggests that the O_h_-form is more stable than the *C*_3v_ one, which is the one observed experimentally (see Scheme 1E). State of the art calculations suggest that both forms are basically isoenergetic (Dixon et al., 2005). The fact that the energies of both structures of XeF_6_ are very close in energy suggests that this molecule is highly fluxional. Therefore, the factors governing the stereoactivity of the lone pair in XeF_6_ are very subtle and, consequently, the lone pair has a highly fluxional character (Dixon et al., 2005).

The interaction energies and equilibrium distances of NgB complexes **25**–**30** are summarized in Table 1. It can be observed that the NgB interaction energies are stronger in XeF_6_ complexes than those in XeF_2_ and XeF_4_ complexes, as predicted by the MEP analysis. Table 1 shows that complexes **25** (X = CO), **26** (X = HF) and **27** (X = HCN) are the weakest ones and exhibit equilibrium distances that are significantly longer than ΣR_cov_ and shorter than ΣR_vdw_, thus suggesting the non-covalent nature of the interaction. However, the rest of complexes (**28**–**30**) present quite short equilibrium distances, thus anticipating some covalent character in agreement with the strong binding energies.

**Figure 14:**
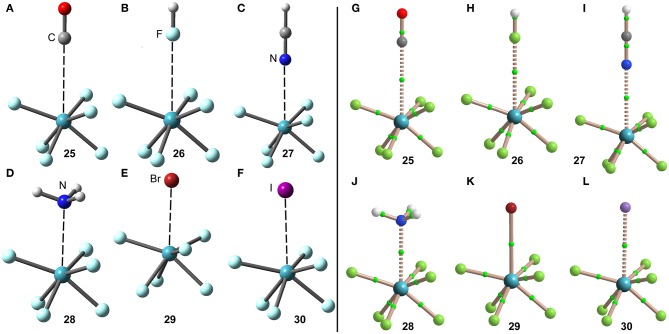
Left panel: PBE1PBE-D3/def2-TZVP Optimized geometries of complexes **25 (A)**, **26 (B)**, **27 (C)**, **28 (D)**, **29 (E)** and **30 (F)**. Distances in Table 1. Right panel: QTAIM distribution of bond critical points (green spheres) and bond paths for complexes **25 (G)**, **26 (H)**, **27 (I)**, **28 (J)**, **28 (K)**, and **30 (L)** at the PBE1PBE-D3/def2-TZVP level of theory.

The optimized geometries of the XeF_6_ complexes are included in Figure 14 (left panel), where electron rich atom is located along the *C*_3_ axis. The symmetry of the XeF_6_ unit in the complexes is *C*_3v_ and the Lewis base is located exactly at the position of the σ-hole represented in Figure 13D. The *C*_3v_-geometry presents a more intense σ-hole thus reinforcing the interaction and compensating the slight deformation energy needed to change from O_h_ to *C*_3v_ that is only 0.33 kcal/mol at the level of theory used herein (PBE1PBE-D3/def2-TZVP). The equilibrium distances range from 2.5 to 3.2 Å (see Table 1), which are shorter compared to the XeF_2_ and XeF_4_ complexes, due to the large σ-hole observed in the XeF_6_ (*C*_3v_-geometry). This behavior is also observed experimentally, since the X-ray structures involving XeF_2_ and XeF_4_ units exhibit significantly longer distances than those of XeF_6_.

The NgB interaction in complexes **25**–**30** has been further characterized using the QTAIM analysis. In agreement with previous observations, the NgB is exclusively characterized by a bond critical point (CP) and bond path that connects the electron rich atom to the Xe atom (see Figure 14, right panel). The values of electron charge density ρ(r) at the bond CPs are tabulated in Table 1 and in line with the rest of complexes, there is good correlation between the values of ρ(r) at the bond CPs that characterize the NgB and the interaction energies by using a logarithmic fitting (regression coefficient, *r* = 0.958, see Supplementary Material). It is interesting to highlight that if all complexes **1**–**30** are used in the same representation, a good relationship is also obtained with a *r* = 0.928 (see ESI). It is worthy to emphasize such relationship, since it allows dealing with all complexes in the same plot. The values of the total energy density [H(r)] at the bond CPs are also summarized in Table 1, which corroborate the non-covalent nature of the interaction in complexes **25**–**27** and partial covalency in complexes **28**–**30**.

The NBO of the XeF_6_···NH_3_ complex has been computed and it shows the typical LP(N)–σ^*^(Xe–F) orbital donor acceptor interaction with an associated stabilization energy of *E*^(2)^ = −13.8 kcal/mol, thus revealing a quite strong orbital contribution. significantly stronger than the other two neutral complexes XeF_2_···NH_3_ and XeF_4_···NH_3_. This contribution is even larger than that in the cationic (XeF_5_···NH_3_)^+^ complex commented above. This result likely explains the short equilibrium distance and large value of charge density at the bond CP in this complex (larger than the iodide complex). It is also worthy to comment that the three NH bonds of the Lewis base are aligned the Xe–F bonds (see Figure 14D), likely contributing to a perfect match between the XeF_6_ and NH_3_ molecules and a shortening of the Xe···N distance.

## Conclusions

From the results reported in this manuscript, the following conclusions arise:

1. There are numerous examples of X-ray structures of XeF_n_ (*n* = 2–6) in the ICSD where non-covalent NgBs play an important role directing the crystal packing and generating interesting supramolecular assemblies, which have been described in detail.
2. The DFT analysis combined with the MEP surfaces show that NgBs are directional and the position of the electron rich atom is determined by the location of the stereo-active lone pair, though the region where the electron pair is located is large and positive.
3. The NgBs in XeF_n_ (*n* = 2, 4, 6) are moderately strong with neutral electron donors and quite strong with anions (and NH_3_ in some cases). Charge assisted NgBs in [XeF_3_]^+^ and [XeF_5_]^+^ cations are very strong and present high covalent character.
4. The NgBs involving xenon fluorides are characterized by a bond CP and bond path interconnecting the xenon to the electron rich atom. The electron charge density at the bond CP can be used as a measure of the strength of the interaction in the whole set of complexes.
5. Orbital donor acceptor charge transfer effects are important contributors to the NgB interactions in the cationic ${\text{XeF}}_{3}^{+}$, ${\text{XeF}}_{4}^{+}$ and also the neutral XeF_6_ molecule, as exemplified by their complexes with NH_3_.

## Data Availability Statement

The raw data supporting the conclusions of this article will be made available by the authors, without undue reservation, to any qualified researcher.

## Author Contributions

RG carried out the theoretical calculations and analyzed the data. AF performed the search in the ICSD database, analyzed the data and wrote the manuscript.

## Conflict of Interest

The authors declare that the research was conducted in the absence of any commercial or financial relationships that could be construed as a potential conflict of interest.

## Supplementary Material

Supplementary Material 1 Logarithmic Regression Plots (Energy in kcal/mol, ρ(r) in a.u.).

Supplementary Material 2 Cartesian coordinates of optimized geometries of all compounds and complexes.

## References

[B1] Adamo C. Barone V. (1999). Toward reliable density functional methods without adjustable parameters: the PBE0 model. J. Chem. Phys. 110, 6158–6170. 10.1063/1.478522

[B2] Bader R. F. W. (1985). Atoms in molecules. Acc. Chem. Res. 18, 9–15. 10.1021/ar00109a003

[B3] Bader R. F. W. (1990). Atoms in Molecules, A Quantum Theory. Clarendon, TX: Oxford.

[B4] Bader R. F. W. Carroll M. T. Cheeseman J. R. Chang C. (1987). Properties of atoms in molecules: atomic volumes. J. Am. Chem. Soc. 109, 7968–7979. 10.1021/ja00260a006

[B5] Bartlett N. Sladky F. O. (1968). The relative fluoride ion donor abilities of XeF_2_, XeF_4_, and XeF_6_ and a chemical purification of XeF_4_. J. Am. Chem. Soc. 90, 5316–5317. 10.1021/ja01021a072

[B6] Bartlett N. (1962). Xenon Hexafluoroplatinate(V) Xe^+^[PtF_6_]^−^. Proc. Chem. Soc. 218–220. 10.1039/PS9620000197

[B7] Bauzá A. Frontera A. (2015). Aerogen bonding interaction: a new supramolecular force? Angew. Chem. Int. Ed. 54, 7340–7343. 10.1002/anie.201502571 25950423

[B8] Bauzá A. Frontera A. (2020). σ/π-Hole noble gas bonding interactions: insights from theory and experiment. Coord. Chem. Rev. 404:213112 10.1016/j.ccr.2019.213112

[B9] Bauzá A. Mooibroek T. J. Frontera A. (2015). The bright future of unconventional σ/π-hole interactions. Chem. Phys. Chem. 16, 2496–2517. 10.1002/cphc.201500314 26118675

[B10] Bauzá A. Seth S. K. Frontera A. (2019). Tetrel bonding interactions at work: impact on tin and lead coordination compounds. Coord. Chem. Rev. 384, 107–125. 10.1016/j.ccr.2019.01.003

[B11] Belpassi L. Infante I. Tarantelli F. Visscher L. (2008). The chemical bond between Au(I) and the noble gases. Comparative study of NgAuF and NgAu+ (Ng = Ar, Kr, Xe) by density functional and coupled cluster methods. J. Am. Chem. Soc. 130, 1048–1060. 10.1021/ja0772647 18161976

[B12] Brock D. S. Mercier H. P. A. Schrobilgen G. J. (2013). [H(OXeF_2_)_n_][AsF_6_] and [FXe^II^(OXe^IV^F_2_)_n_][AsF_6_] (n = 1, 2): examples of Xenon(IV) Hydroxide fluoride and oxide fluoride cations and the crystal structures of [F_3_Xe—FH][Sb_2_F_11_] and [H_5_F_4_][SbF_6_]·2[F_3_Xe—FH][Sb_2_F_11_]. J. Am. Chem. Soc. 135, 5089–5104. 10.1021/ja312493j 23398504

[B13] Burns J. H. Ellison R. D. Levy H. A. (1965). The crystal structure of the molecular addition compound xenon difluoride–xenon tetrafluoride. Acta Crystallogr. 18, 11–16. 10.1107/S0365110X65000038

[B14] Busschaert N. Caltagirone C. Rossom W. van Gale P. A. (2015). Applications of supramolecular anion recognition. Chem. Rev. 115, 8038–8155. 10.1021/acs.chemrev.5b00099 25996028

[B15] Cavallo G. Metrangolo P. Milani R. Pilati T. Priimagi A. Resnati G.. (2016). The halogen bond. Chem. Rev. 116, 2478–2601. 10.1021/acs.chemrev.5b00484 26812185 PMC4768247

[B16] Chernick C. L. Claassen H. H. Fields P. R. Hyman H. H. Malm J. G. Manning W. M.. (1962). Fluorine compounds of xenon and radon. Science 138, 136–138. 10.1126/science.138.3537.136 17818399

[B17] Christe K. O. Curtis E. C. Dixon D. A. Mercier H. P. A. Sanders J. C. P. Schrobilgen G. J. (1991). Crystal structures of [xenon fluoride(+)][ruthenium hexafluoride(–)] and [xenon pentafluoride(+)][ruthenium hexafluoride(–)]. J. Am. Chem. Soc. 113, 3351–3361. 10.1021/ja00009a021

[B18] Cohen B. Peacock R. D. (1966). Properties of xenon fluoride adducts. J. Inorg. Nucl. Chem. 28, 3056–3057. 10.1016/0022-1902(66)80037-4

[B19] Cooke S. A. Gerry M. C. L. (2004). XeAuF. J. Am. Chem. Soc. 126, 17000–17008. 10.1021/ja044955j 15612738

[B20] Desiraju G. R. (2013). Crystal engineering: from molecule to crystal. J. Am. Chem. Soc. 135, 9952–9967. 10.1021/ja403264c 23750552

[B21] Desiraju G. R. Steiner T. (2001). The Weak Hydrogen Bond in Structural Chemistry and Biology. Oxford: Oxford University Press.

[B22] Dixon D. A. de Jong W. A. Peterson K. A. Christe K. O. Schrobilgen G. J. (2005). Heats of formation of xenon fluorides and the fluxionality of XeF_6_ from high level electronic structure calculations. J. Am. Chem. Soc. 127, 8627–8634. 10.1021/ja0423116 15954767

[B23] Edwards A. J. Holloway J. H. Peacock R. D. (1963). New fluorine compounds of xenon. Proc. Chem. Soc. 275–276. 10.1039/ps9630000253

[B24] Frisch M. J. Trucks G. W. Schlegel H. B. Scuseria G. E. Robb M. A. Cheeseman J. R. (2016). Gaussian 16, Revision B.01. Wallingford, CT: Gaussian Inc.

[B25] Frontera A. Gamez P. Mascal M. Mooibroek T. J. Reedijk J. (2011). Putting anion– π interactions into perspective. Angew. Chem. Int. Ed. 50, 9564–9583. 10.1002/anie.201100208 21928463

[B26] Gawrilow M. Beckers H. Riedel S. Cheng L. (2018). Matrix–isolation and quantum–chemical analysis of the C_3v_ conformer of XeF_6_, XeOF_4_, and their acetonitrile adducts. J. Phys. Chem. A 122, 119–129. 10.1021/acs.jpca.7b09902 29220184

[B27] Gillespie R. J. Martin D. Schrobilgen G. J. Slim D. R. (1977). The crystal structure of trifluoroxenon(IV) hexafluorobismuthate(V): the fluoride–acceptor strength of bismuth pentafluoride. J. Chem. Soc. Dalton Trans. 2234–2237. 10.1039/dt9770002234

[B28] Gillespie R. J. Pez G. P. (1969). Fluorosulfuric acid solvent system. VII. The behavior of some extremely weak bases in the superacid system fluorosulfuric acid– antimony pentafluoride–sulfur trioxide. Inorg. Chem. 8, 1233–1235. 10.1021/ic50076a006

[B29] Grandinetti F. (2018). Noble Gas Chemistry: Structure, Bonding, and Gas–Phase Chemistry. Weinheim: Wiley–VCH.

[B30] Grochala W. (2007). Atypical compounds of gases, which have been called ‘noble'. Chem. Soc. Rev. 36, 1632–1655. 10.1039/b702109g 17721587

[B31] Hagiwara R. Hollander F. Maines C. Bartlett N. (1991). The crystal–structure of [Ag(XeF_2_)_2_]AsF_6_ formed in the oxidation of Xe by AgFAsF_6_. Eur. J. Solid State Chem. 28, 855–866.

[B32] Haner J. Matsumoto K. Mercier H. P. A. Schrobilgen G. J. (2016). Nature of the Xe^VI^-N bonds in F_6_XeNCCH_3_ and F_6_Xe(NCCH_3_)_2_ and the stereochemical activity of their xenon valence electron lone pairs. Chem. Eur. J. 22, 4833–4842. 10.1002/chem.201504904 26918266

[B33] Haner J. Schrobilgen G. J. (2015). The chemistry of xenon(IV). Chem. Rev. 115, 1255–1295. 10.1021/cr500427p 25559700

[B34] Holloway J. H. (1968). Noble–Gas Chemistry. London: Methuen.

[B35] Hoyer S. Emmler T. Seppelt K. (2006). The structure of xenon hexafluoride in the solid state. J. Fluorine Chem. 127, 1415–1422. 10.1016/j.jfluchem.2006.04.014

[B36] Hughes M. J. Brock D. S. Mercier H. P. A. Schrobilgen G. J. (2011). A Raman spectroscopic study of the XeOF_4_/XeF_2_ system and the X–ray crystal structure of α− XeOF_4_·XeF_2_. J. Fluorine Chem. 132:660 10.1016/j.jfluchem.2011.05.010

[B37] Ibers J. A. Hamilton W. C. (1963). Xenon tetrafluoride: crystal structure. Science 139, 106–107. 10.1126/science.139.3550.106 17798707

[B38] Jones G. R. Burbank R. D. Bartlett N. (1970). The crystal structure of the 1:1 molecular addition compound xenon difluoride–iodine pentafluoride, XeF_2_·IF_5_. Inorg. Chem. 9, 2264 10.1021/ic50092a011

[B39] Kaupp M. van Wüllen Ch. Franke R. Schmitz F. Kutzelnigg W. (1996). The structure of XeF_6_ and of compounds isoelectronic with it. A challenge to computational chemistry and to the qualitative theory of the chemical bond. J. Am. Chem. Soc. 118, 11939–11950. 10.1021/ja9621556

[B40] Keith T. A. (2013). AIMAll (Version 13.05.06), TK Gristmill Software. Kansas City, KS.

[B41] Koppe K. Haner J. Mercier H. P. A. Frohn H.–J. Schrobilgen G. J. (2014). Xenon(IV)–carbon bond of [C_6_F_5_XeF_2_]^+^; structural characterization and bonding of [C_6_F_5_XeF_2_][BF_4_], [C_6_F_5_XeF_2_][BF_4_]·2HF, and [C_6_F_5_XeF_2_][BF_4_]·*n*NCCH 3 (*n* = 1, 2); and the fluorinating properties of [C_6_F_5_XeF_2_][BF_4_]. Inorg. Chem. 53, 11640–11661. 10.1021/ic501831j 25330056

[B42] Legon A. C. (2017). Tetrel, pnictogen and chalcogen bonds identified in the gas phase before they had names: a systematic look at non–covalent interactions. Phys. Chem. Chem. Phys. 19, 14884–14896. 10.1039/C7CP02518A 28561824

[B43] Lutar K. Borrmann H. Žemva B. (1998). XeF_2_·2CrF_4_and ${\text{XeF}}_{5}^{+}$${\text{CrF}}_{5}^{-}$: syntheses, crystal structures, and some properties. Inorg. Chem. 37, 3002–3006. 10.1021/ic971580c

[B44] Lutar K. Leban I. Ogrin T. Zemva B. (1992). ${\text{XeF}}_{2}^{*}$CrF_4_ and [XeF_5_(+)CrF_5_${\left(–)\right]}_{4}^{*}$XeF_4_: syntheses, crystal structures and some properties. Eur. J. Solid State Inorg. Chem. 29, 713–727. 10.1002/chin.199836008

[B45] Matsumoto K. Haner J. Mercier H. P. A. Schrobilgen G. J. (2015). Syntheses and structures of F_6_XeNCCH_3_ and F_6_Xe(NCCH_3_)_2_. Angew. Chem., Int. Ed. 54, 14169–14173. 10.1002/anie.201507635 26388107

[B46] McKee D. E. Zalkin A. Bartlett N. (1973). Crystal structure of [${\text{XeF}}_{3}^{+}$][Sb_2_${\text{F}}_{11}^{-}$]. Inorg. Chem. 12, 1713–1717. 10.1021/ic50126a001

[B47] Meyer E. A. Castellano R. K. Diederich F. (2003). Interactions with aromatic rings in chemical and biological recognition. Angew. Chem. Int. Ed. 42, 1210–1250. 10.1002/anie.200390319 12645054

[B48] Munárriz J. Calatayud M. Contreras-García J. (2019). Valence-shell electron-pair repulsion theory revisited: an explanation for core polarization. Chem. Eur. J. 25, 10938–10945. 10.1002/chem.201902244 31206860

[B49] Reed A. E. Curtiss L. A. Weinhold F. (1988). Intermolecular interactions from a natural bond orbital, donor–acceptor viewpoint. Chem. Rev. 88, 899–926. 10.1021/cr00088a005

[B50] Scheiner S. (2013). The pnicogen bond: its relation to hydrogen, halogen, and other noncovalent bonds. Acc. Chem. Res. 46, 280–288. 10.1021/ar3001316 23135342

[B51] Schneider H.–J. Yatsimirski A. (2000). Principles and Methods in Supramolecular Chemistry. Chichester: Wiley.

[B52] Schneider H. J. (2009). Binding mechanisms in supramolecular complexes. Angew. Chem. Int. Ed. 48, 3924–3977. 10.1002/anie.200802947 19415701

[B53] Scilabra P. Terraneo G. Resnati G. (2019). The chalcogen bond in crystalline solids: a world parallel to halogen bond. Acc. Chem. Res. 52, 1313–1324. 10.1021/acs.accounts.9b00037 31082186

[B54] Seidel S. Seppelt K. (2000). Xenon as a complex ligand: the tetra xenono Gold(II) cation in ${\text{AuXe}}_{4}^{2+}$(Sb_2_${\text{F}}_{11}^{-}$)_2_. Science 290, 117–118. 10.1126/science.290.5489.117 11021792

[B55] Seppelt K. (2015). Molecular hexafluorides. Chem. Rev. 115, 1296–1306. 10.1021/cr5001783 25418862

[B56] Sladky F. O. Bulliner P. A. Bartlett N. (1969). Xenon difluoride as a fluoride ion donor. evidence for the salts [Xe_2_F_3_]^+^[MF_6_]^−^,[XeF]^+^[MF_6_]^−^ and [XeF]^+^[M_2_F_11_]^−^. J. Chem. Soc. A 2179–2188. 10.1039/J19690002179

[B57] Tavčar G. Tramšek M. (2015). XeF_2_ as a ligand to a metal center, an interesting field of noble gas chemistry. J. Fluorine Chem. 174, 14–21. 10.1016/j.jfluchem.2014.08.009

[B58] Tavčar G. Tramšek M. Bunič T. Benkič P. Žemva B. (2004). New class of coordination compounds with noble gas fluorides as ligands to metal ions. J. Fluorine Chem. 125, 1579–1584. 10.1016/j.jfluchem.2004.08.006

[B59] Tavčar G. Žemva B. (2009). XeF_4_ as a ligand for a metal ion. Angew. Chem. Int. Ed. 48, 1432–1434. 10.1002/anie.200803365 19140146

[B60] Templeton D. H. Zalkin A. Forrester J. D. Williamson S. M. (1963). Crystal and molecular structure of xenon tetrafluoride. J. Am. Chem. Soc. 85, 242–242. 10.1021/ja00885a038

[B61] Tramšek M. Benkič P. Žemva B. (2002). [M(XeF_2_)_3_](AsF_6_)_2_ (M=Pb, Sr): the first coordination compounds of M^2+^ with XeF_2_ ligand. Solid State Sci. 4, 9–14. 10.1016/S1293-2558(01)01206-7

[B62] Tramšek M. Žemva B. (2006). Synthesis of novel salts with HF, AsF_3_ and XeF_2_ as ligands to metal cations. J. Fluorine Chem. 127, 1275–1284. 10.1016/j.jfluchem.2006.05.014

[B63] Weigend F. (2006). Accurate coulomb–fitting basis sets for H to Rn. Phys. Chem. Chem. Phys. 8, 1057–1065. 10.1039/b515623h 16633586

[B64] Weigend F. Ahlrichs R. (2005). Balanced basis set of split valence, triple zeta valence and quadrupole zeta valence quality for H to Rn: design and assessment of accuracy. Phys. Chem. Chem. Phys. 7, 3297–3305. 10.1039/b508541a 16240044

[B65] Zhao L. Pan S. Holzmann N. Schwerdtfeger P. Frenking G. (2019). Chemical bonding and bonding models of main–group compounds. Chem. Rev. 119, 8781–8845. 10.1021/acs.chemrev.8b00722 31251603

[B66] Zhao Y. Benz S. Sakai N. Matile S. (2015). Selective acceleration of disfavored enolate addition reactions by anion–π interactions. Chem. Sci. 6, 6219–6223. 10.1039/C5SC02563J 30090238 PMC6054047

