# Supplementary material for: Covalent and Non-covalent Noble Gas Bonding Interactions in XeFn Derivatives (n = 2–6): A Combined Theoretical and ICSD Analysis
Source: Front Chem. 2020 May 6;8:395. doi: 10.3389/fchem.2020.00395 (PMC7218167; doi:10.3389/fchem.2020.00395)
Supplement: Supplementary Material 1 — Logarithmic Regression Plots (Energy in kcal/mol, ρ(r) in a.u.). [file Data_Sheet_1.docx]

Supplementary Material

# Logarithmic Regression Plots (Energy in kcal/mol, ρ(r) in a.u.)

# Cartesian Coordinates

**1** (XeF_2_··CO)

54 -0.000000 0.000000 0.727902

9 -0.000000 1.981141 0.728503

9 -0.000000 -1.981141 0.728503

8 0.000000 0.000000 -4.225355

6 0.000000 0.000000 -3.102820

**2** (XeF_2_··FH)

54 0.000000 0.000000 0.416231

9 0.000000 1.982218 0.428009

9 -0.000000 -1.982218 0.428009

9 0.000000 0.000000 -2.926012

1 0.000000 0.000000 -3.846539

**3** (XeF_2_··NCH)

54 0.000000 -0.000000 0.680038

9 0.000000 1.983019 0.694177

9 -0.000000 -1.983019 0.694177

6 0.000000 0.000000 -4.011632

1 0.000000 0.000000 -5.080781

7 0.000000 0.000000 -2.866669

**4** (XeF_2_··NH3)

54 0.000000 0.415637 0.000000

9 -1.702193 -0.611965 0.000000

9 1.663171 1.495374 0.000000

7 0.094994 -2.943732 0.000000

1 -0.509594 -3.018717 0.809173

1 0.705425 -3.751524 0.000000

1 -0.509594 -3.018717 -0.809173

**5** (XeF_2_··Br) ^–^

54 -0.000000 1.100900 -0.000000

9 1.923358 0.596260 -0.000000

9 -1.872041 1.903617 -0.000000

35 -0.013196 -2.341357 0.000000

**6** (XeF_2_··I) ^–^

54 -0.000000 1.575007 0.000000

9 -1.885570 2.325124 0.000000

9 1.924051 1.078484 0.000000

53 -0.006535 -2.182695 -0.000000

**7** (XeF_3_··CO)^+^

54 0.000000 0.294009 -0.000000

9 1.892333 0.392109 -0.000000

9 0.023929 2.141047 -0.000000

9 -1.889224 0.440217 -0.000000

6 -0.014859 -2.410129 0.000000

8 -0.019274 -3.522009 0.000000

**8** (XeF_3_··FH)^+^

54 0.000000 0.083232 0.000000

9 1.524746 -1.025135 0.000000

9 1.233134 1.443300 0.000000

9 -1.257692 1.484677 0.000000

9 -1.358297 -2.069083 0.000000

1 -1.277018 -2.998348 0.000000

**9** (XeF_3_··NCH)^+^

54 0.000000 0.258834 -0.000000

9 -1.898787 0.367343 -0.000000

9 0.000663 2.110111 -0.000000

9 1.898868 0.366003 -0.000000

6 -0.000283 -3.319300 0.000000

1 0.000251 -4.396557 0.000000

7 -0.000751 -2.179397 0.000000

**10** (XeF_3_··NH_3_)^+^

54 0.000169 0.107650 0.000000

9 0.000169 -0.026594 1.919763

9 -0.027107 1.998555 0.000000

9 0.000169 -0.026594 -1.919763

7 0.023044 -2.229529 -0.000000

1 0.981554 -2.578683 -0.000000

1 -0.455515 -2.568003 -0.835602

1 -0.455515 -2.568003 0.835602

**11** (XeF_3_··Br)

54 0.000000 0.614749 0.000000

9 1.950399 0.645566 0.000000

9 0.000809 2.598584 0.000000

9 -1.950519 0.645491 0.000000

35 -0.000177 -1.948662 0.000000

**12** (XeF_3_··I)

54 0.000000 0.961703 0.000000

9 1.956306 0.971087 0.000000

9 0.002764 2.973536 0.000000

9 -1.956682 0.971365 0.000000

53 -0.000406 -1.814639 0.000000

**13** (XeF_4_··CO)

54 0.549123 0.000015 0.090694

9 1.568612 1.367116 -0.836984

9 -0.451908 1.378822 1.021061

9 -0.463073 -1.363313 1.031795

9 1.556850 -1.382634 -0.826635

8 -4.015690 0.000420 -0.536210

6 -2.903575 -0.000681 -0.685151

**14** (XeF_4_··FH)

54 0.248035 0.195434 0.000000

9 0.261340 1.558496 1.379733

9 0.261340 -1.200431 1.355847

9 0.261340 -1.200431 -1.355847

9 0.261340 1.558496 -1.379733

9 -2.330451 -1.622460 -0.000000

1 -1.828087 -2.396449 -0.000000

**15** (XeF_4_··NCH)

54 -0.137018 -0.202779 -0.000000

9 -0.383435 -1.493627 1.310474

9 -0.383435 1.089224 1.292438

9 -0.383435 1.089224 -1.292438

9 -0.383435 -1.493627 -1.310474

6 3.191538 1.685466 0.000000

1 4.115123 2.235594 0.000000

7 2.210217 1.102349 0.000000

9 -1.948141 -0.203949 -0.000000

**16** (XeF_4_··NH_3_)

54 0.259761 0.179342 -0.000000

9 0.289966 1.551486 1.383767

9 0.289966 -1.191572 1.377769

9 0.289966 -1.191572 -1.377769

9 0.289966 1.551486 -1.383767

7 -2.375563 -1.530143 0.000000

1 -1.888767 -2.418593 0.000000

1 -2.974081 -1.516647 -0.816775

1 -2.974081 -1.516647 0.816775

**17** (XeF_4_··Br) ^–^

54 0.016250 0.875007 -0.000000

9 -0.014032 0.935436 1.962059

9 -1.865162 0.365927 -0.000000

9 -0.014032 0.935436 -1.962059

9 1.850294 1.702241 -0.000000

35 -0.014032 -2.362907 0.000000

**18** (XeF_4_··I) ^–^

54 -0.015133 1.284289 0.000000

9 1.863218 0.759222 0.000000

9 0.010668 1.338924 1.960048

9 -1.856575 2.083754 0.000000

9 0.010668 1.338924 -1.960048

53 0.010668 -2.246019 -0.000000

**19** (XeF_5_··CO)^+^

54 0.271048 0.000034 -0.098262

9 1.191744 1.292602 -1.044267

9 -0.253826 1.302263 1.094305

9 -0.266416 -1.278950 1.113643

9 1.177242 -1.316416 -1.024987

8 -3.848080 -0.000491 -0.303733

6 -2.733434 0.001308 -0.290646

9 1.767772 -0.000139 0.914627

**20** (XeF_5_··FH)^+^

54 -0.000969 -0.110413 -0.000000

9 -0.236826 -1.400693 1.295009

9 -0.236826 1.179509 1.290718

9 -0.236826 1.179509 -1.290718

9 -0.236826 -1.400693 -1.295009

9 2.420213 1.027127 0.000000

1 3.063085 1.698948 0.000000

9 -1.807440 -0.111054 -0.000000

**21** (XeF_5_··NCH)^+^

54 -0.093743 0.212423 -0.000000

9 1.697294 -0.223245 -0.000000

9 0.009013 0.406362 1.838251

9 -1.707109 1.133987 -0.000000

9 0.573611 1.895299 -0.000000

9 0.009013 0.406362 -1.838251

6 0.009013 -3.639301 0.000000

1 0.056871 -4.713240 0.000000

7 -0.040748 -2.498668 0.000000

**22** (XeF_5_··NH_3_)^+^

54 0.015711 -0.133364 -0.000000

9 -0.223856 -1.407976 1.356661

9 -0.223856 1.178693 1.289360

9 -0.223856 1.178693 -1.289360

9 -0.223856 -1.407976 -1.356661

7 2.254404 1.180856 0.000000

1 2.030613 2.173839 0.000000

1 2.826166 0.995612 -0.821362

1 2.826166 0.995612 0.821362

9 -1.805928 -0.122477 -0.000000

**23** (XeF_5_··Br)

54 -0.520423 0.000351 0.000000

9 -0.611956 -1.362150 1.358460

9 -0.611956 -1.362150 -1.358460

9 -0.611956 1.360194 -1.363587

9 -2.459559 0.000160 0.000000

9 -0.611956 1.360194 1.363587

35 2.064836 0.000423 0.000000

**24** (XeF_5_··I)

54 -0.846256 -0.000268 0.000000

9 -0.898587 -1.366420 1.365771

9 -0.898587 -1.366420 -1.365771

9 -0.898587 1.365644 -1.366268

9 -2.819954 -0.000362 0.000000

9 -0.898587 1.365644 1.366268

53 1.951444 0.000598 0.000000

**25** (XeF6··CO)

54 0.000000 0.000000 0.317973

9 0.000000 1.843738 -0.359793

9 1.596724 -0.921869 -0.359793

9 -1.596724 -0.921869 -0.359793

9 0.000000 -1.420434 1.530407

9 -1.230132 0.710217 1.530407

9 1.230132 0.710217 1.530407

8 0.000000 0.000000 -3.964224

6 0.000000 0.000000 -2.843882

**26** (XeF6··FH)

54 0.000000 0.000000 0.134993

9 0.000000 1.840757 -0.538501

9 1.594142 -0.920379 -0.538501

9 -1.594142 -0.920379 -0.538501

9 0.000000 -1.419181 1.350323

9 -1.229047 0.709590 1.350323

9 1.229047 0.709590 1.350323

9 0.000000 0.000000 -2.828763

1 0.000000 0.000000 -3.749945

**27** (XeF6··NCH)

54 0.000000 0.000000 0.272500

9 -0.000000 1.860593 -0.358543

9 1.611321 -0.930296 -0.358543

9 -1.611321 -0.930296 -0.358543

9 0.000000 -1.415315 1.495894

9 -1.225699 0.707657 1.495894

9 1.225699 0.707657 1.495894

6 0.000000 0.000000 -3.739098

1 0.000000 0.000000 -4.808597

7 0.000000 0.000000 -2.597183

**28** (XeF6··NH3)

54 0.000000 0.000000 0.089557

9 0.000000 1.883274 -0.534021

9 1.630963 -0.941637 -0.534021

9 -1.630963 -0.941637 -0.534021

9 0.000000 -1.433701 1.315999

9 -1.241622 0.716851 1.315999

9 1.241622 0.716851 1.315999

7 0.000000 0.000000 -2.496492

1 -0.829526 -0.478927 -2.824681

1 0.829526 -0.478927 -2.824681

1 0.000000 0.957854 -2.824681

**29** (XeF6··Br)^–^

54 0.000000 0.000000 0.534889

9 -0.000000 1.930457 0.056049

9 -1.268808 0.732547 1.818876

9 -1.671825 -0.965229 0.056049

9 -0.000000 -1.465094 1.818876

9 1.671825 -0.965229 0.056049

9 1.268808 0.732547 1.818876

35 0.000000 0.000000 -2.271629

**30** (XeF6··I)^–^

54 0.000000 0.000000 0.872430

9 0.000000 1.927987 0.380696

9 -1.278831 0.738334 2.149573

9 -1.669686 -0.963994 0.380696

9 -0.000000 -1.476667 2.149573

9 1.669686 -0.963994 0.380696

9 1.278831 0.738334 2.149573

53 0.000000 0.000000 -2.177895
